# Myopia, Sodium Chloride, and Vitreous Fluid Imbalance: A Nutritional Epidemiology Perspective

**DOI:** 10.3390/epidemiologia5010003

**Published:** 2024-01-29

**Authors:** Ronald B. Brown

**Affiliations:** School of Public Health Sciences, University of Waterloo, Waterloo, ON N2L 3G1, Canada; r26brown@uwaterloo.ca

**Keywords:** axial myopia, sodium chloride, vitreous humor, nutritional epidemiology, grounded theory, osmolality, low-salt diet

## Abstract

Theories of myopia etiology based on near work and lack of outdoor exposure have had inconsistent support and have not prevented the rising prevalence of global myopia. New scientific theories in the cause and prevention of myopia are needed. Myopia prevalence is low in native people consuming traditional diets lacking in sodium chloride, and nutritional epidemiological evidence supports the association of rising myopia prevalence with dietary sodium intake. East Asian populations have among the highest rates of myopia associated with high dietary sodium. Similar associations of sodium and rising myopia prevalence were observed in the United States in the late 20th century. The present perspective synthesizes nutritional epidemiology evidence with pathophysiological concepts and proposes that axial myopia occurs from increased fluid retention in the vitreous of the eye, induced by dietary sodium chloride intake. Salt disturbs ionic permeability of retinal membranes, increases the osmotic gradient flow of fluid into the vitreous, and stretches ocular tissue during axial elongation. Based on the present nutritional epidemiology evidence, experimental research should investigate the effect of sodium chloride as the cause of myopia, and clinical research should test a very low-salt diet in myopia correction and prevention.

## 1. Introduction

In a 2005 study of native Amazon tribes of northwestern Brazil, Thorn et al. noted that most of the people in the study were illiterate and “remarkably free of myopia” [1]. The researchers implied an association of nearsightedness with near work (e.g., reading), but more recent epidemiological findings linking near work with myopia are inconsistent [2]. Other theories have associated myopia with environmental factors, such as “use of mobile devices, spending more time indoors or less time outdoors, or lack of exposure to sunshine”, yet researchers note that a consensus has not been reached confirming the causality of these factors [3]. Notably, among the Amazon tribes whom Thorn et al. described as “remarkably free of myopia”, the traditional diet of the Yanomamo tribe is also remarkably free of sodium chloride [4].

Similarly, artic explorer Vilhjalmur Stefansson noted the absence of dietary salt in the traditional diet of indigenous people within the Artic regions, “in whose language the word *mamaitok*, meaning ‘salty’, is synonymous with ‘evil-tasting’” [5]. In 1969, Young et al. reported that myopia prevalence was low among older indigenous people of Artic regions who consumed a traditional diet, compared to an unusually high 58% myopia prevalence among indigenous offspring [6]. Increased myopia in Artic regions parallels a transition to a salty Western diet and the consumption of “processed and packaged products” [7]. Recently, refractive errors have reached 78% prevalence among northern Canadian indigenous children [8].

Studying the beneficial effects of diet on hypertension, Dr. Walter Kempner published findings in 1948 describing how patients consuming a no-salt diet of rice and fruit reversed advanced retinopathy [9], a degenerative condition associated with severe myopia [10]. Follow-up experimental studies based on Kempner’s clinical findings were conducted in 1948 by Stocker, Holt, and Clower [11]. The researchers found that patients without glaucoma who followed the no-salt rice diet “showed a striking and persistent reduction of intraocular tension.” No correlation was found between overall reductions in arterial blood pressure and ocular pressure, prompting the researchers to attribute the reduction in ocular pressure to lowered levels of sodium and chloride ions within the eye. Stocker et al. explained how the flow of fluid and electrolytes, ions formed from minerals derived from dietary sources, influence the mechanics of the eye.

However, since the findings of Kempner and Stocker et al. in the late 1940s, “relatively little attention has been paid to the possible role of nutrition in myopia” [12]. In particular, dietary salt restriction was ignored for decades as an effective and inexpensive treatment to reduce hypertension and to lower the effects of related ocular pathologies [13]. Moreover, as of 2023, “there are numerous conflicting findings, and no definitive consensus has been reached on the impact of diet on the occurrence and progression of myopia” [14]. For example, Chua et al. found that dietary assessments of protein, fat, carbohydrate, and energy in Singaporean children were not associated with the development of myopia at three years of age [15] and nine years of age [16], yet dietary sodium is not mentioned in either study.

A 2023 systematic review on myopia and nutrition found that “most of the nutrients and dietary elements investigated in noninterventional studies showed inconsistencies in their association with myopia, with the majority indicating no association” [17], but none of the studies in the systematic review investigated dietary sodium or salt associated with myopia. New scientific theories based on nutritional epidemiology and pathophysiological evidence are needed for future research in the cause and prevention of myopia. 

Nutritional epidemiology investigates dietary factors associated with disease risks within a population. The present perspective paper used a grounded theory literature review method described by Wolfswinkel et al. [18] to select, compare, and analyze evidence from nutritional epidemiology, ocular pathophysiology, and experimental research literature on myopia, dietary sodium and salt, and vitreous fluid imbalance. A rigorous and objective review of the scientific evidence in the present paper supports a novel theory positing that axial myopia is associated with fluid imbalance in ocular tissue induced by dietary sodium chloride. Findings presented in this paper can help guide directions for future clinical and experimental research to verify the pathophysiological mechanisms of salt intake with myopia.

## 2. Myopia History and Pathophysiology

Descriptions of eye blurriness appeared around 1550 BC in ancient Egypt [19], and the ancient Egyptians had discovered how to preserve meat with salt [20]. “Blindness and varying degrees of visual impairment were widespread in the ancient Greco-Roman world” [21], and Roman emperor Nero was said to be nearsighted [22]. Salt was a highly valued commodity at the time, and the ancient Greeks described the value of a slave with the expression, “not worth his salt”, while the word “salary” is derived from the salt paid to Roman soldiers [23]. In ancient China, salt was used to preserve food as early as 5000 years ago [23], and “interest in diseases of the eyes (which were probably rampant in antiquity) is evident in early medical writings from the Middle East, India and China” [24]. Greek philosopher Aristotle is credited with coining the word myopia in 350 BC [22]. In his Book of Problems, Aristotle wrote, “the eye is moist above all parts of the body, and of a waterish nature; and as the water is clear and smooth, so likewise is the eye” [25].

**Figure 1:**
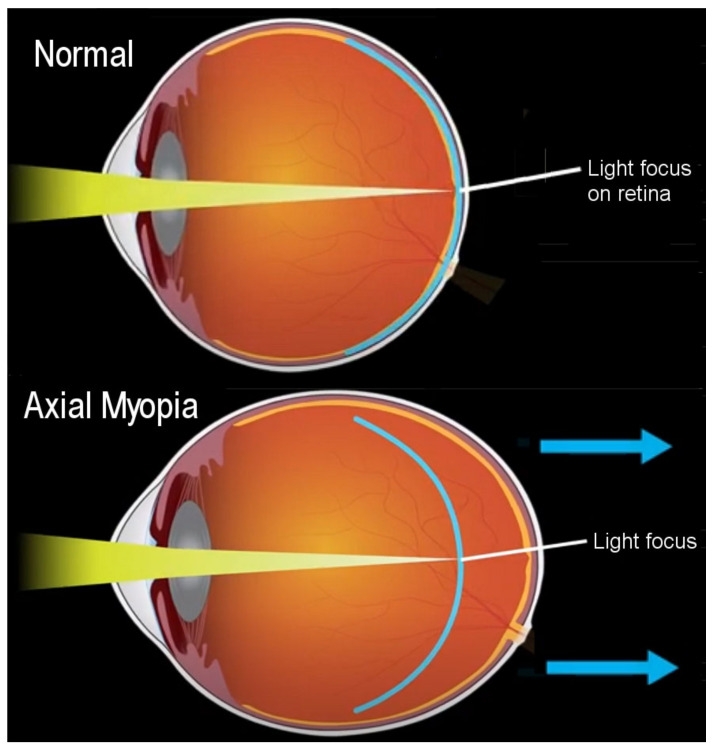
Normal eye and myopia, based on the National Eye Institute, National Institutes of Health (NIE/NIH) [29]. The blue arrows indicate the direction of axial elongation.

Refractive errors in myopia are categorized as mild (−0.5 to −4 diopters), moderate (−4 to −8 diopters), and severe high (>−8 diopters) [26]. The risk of eye diseases, such as glaucoma, cataract, retinal detachment, and myopic macular degeneration, increases in high myopia [27]. Among people over 70 years of age with high myopia, pathological myopia prevalence is 65%, a leading cause of blindness worldwide [28]. Axial myopia, the most common type of myopia or nearsightedness, is caused by the increased axial length of the eye, which increases the distance between the retina in the posterior segment of the eye and the focus of light coming through the cornea and lens, as shown in Figure 1 [29]. The retina is part of the central nervous system and transforms light into electrical impulses, which are transmitted to the optic nerve. Less commonly, refractive myopia shortens the focus of light in front of the retina, due to structural or location changes in the cornea and lens [30]. Interestingly, Figure 1 shows that the length of focused light in axial myopia is generally comparable to normal vision, implicating the displaced location of the retina due to eye elongation, indicated by the blue arrows in the figure. The retina lines the vitreous humor in the posterior chamber, which is filled with a gel and water that provides shape to the eye. Logically, changes in the ocular fluid volume are potentially related to axial elongation in myopia.

## 3. Nutritional Epidemiology of Myopia and Dietary Sodium

Myopia is projected to affect about half of the global population (49.8%) by 2050 [31], and reducing myopia prevalence is a “high research priority” [30]. Global annual loss of productivity from uncorrected vision impairment in myopia was estimated at USD 244 billion in 2015 [32], and higher myopic refractive error in patients tends to adversely affect quality of life [27]. Although asthenopia (visual fatigue) may accompany myopia, studies show that asthenopia can occur without refractive errors [33].

Myopia is associated with genetic factors, and “more than 400 associated gene loci have been mapped for myopia and refractive errors” [34]. Additionally, a strong role is played by environmental and lifestyle factors. For example, the prevalence of myopia in immigrant children arriving in Israel at an older age was lower than in children born in Israel or children immigrating at an earlier age [35]. Migrant studies demonstrate how health in people immigrating to a new country can decline as immigrants adjust to life in their adopted country, “perhaps caused by changes in nutrition, customs and lifestyle” and other factors [36].

Lower prevalence of myopia in Chinese children and adolescents was associated with longer physical activity duration, suggested to be linked to exposure to outdoor sunlight [37]. Additionally, physical activity can lower sodium levels through perspiration, and future studies should investigate how sodium and fluid loss during physical activity affect fluid balance in the vitreous of the eye in myopia. High dietary sodium intake is also an established risk factor for high blood pressure [38], and a study in Taiwan found a significant association between high systolic blood pressure and myopic maculopathy in participants 65 years of age and older, inferring that high dietary sodium potentially mediates this association. Another population study analyzing 24 h urinary sodium excretion found that East Asian countries are also among the highest consumers of dietary sodium [39]. Interestingly, the current myopia prevalence in urban areas of East Asian countries has risen to 80–90% among young adults, and myopia is prevalent in 96.5% of 19-year-old men in Seoul, South Korea [40]. 

**Figure 2:**
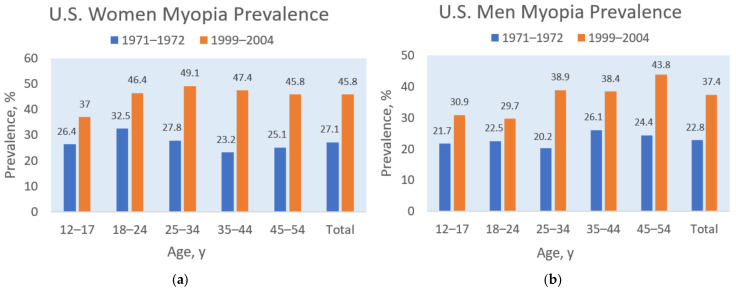
Myopia prevalence for women (**a**) and men (**b**) by age groups in the 19711972 and 1999–2004 U.S. National Health and Nutrition Examination Surveys, based on Vitale et al. [41].

**Figure 3:**
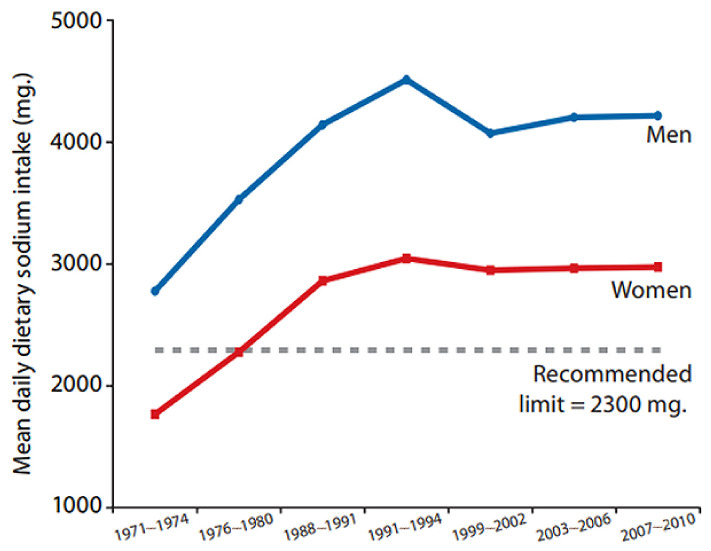
Mean daily dietary sodium intake in U.S. men and women aged 18–74 in the 1971 to 2010 National Health and Nutrition Examination Surveys [42]. Courtesy of New York State Department of Health.

A similar relationship between dietary sodium and myopia prevalence appeared in the United States. For example, Figure 2 shows that myopia prevalence increased in the U.S. population during the last three decades of the 20th century, rising, on average, from 27.1% to 45.8% in women and from 22.8% to 37.4% in men [41]. Simultaneously, dietary sodium intake increased in the adult U.S. population during the same period, as shown in Figure 3 [42]. A possible mediating factor explaining the association of rising myopia prevalence with increasing salt intake could be the rapid increase in consumption of ultra-processed food in North America, rising in Canada from “24 to 61.7% of total calories” during the late 20th century [43]. Increased consumption of ultra-processed food with added sodium, sugar, and fat could also mediate the rising prevalence of myopia associated with increasing rates of obesity [35,44], and more research is needed in this area.

More recently, global daily sodium intake averages 4310 mg (just over 10 g salt—sodium comprises approximately 40% of sodium chloride by weight), and the World Health Organization (WHO) recommends that adults consume less than 2000 mg of sodium or under 5 g of salt daily [45]. Moreover, the National Heart, Lung, and Blood Institute of the U.S. National Institutes of Health recommends 500 mg of sodium to meet daily basal requirements [46], which can be obtained from natural whole foods without added salt. Dr. Walter Kempner obtained clinical benefits in patients following his fruit and rice diet with only 150 mg of sodium per 2000 calories a day [9], and Campbell estimated the daily sodium intake of the Yanomamo tribe of the Amazon at less than 100 mg [47].

Myopia prevalence is also common in diseases and illnesses associated with a high intake of sodium chloride, such as migraine and multiple sclerosis (MS). Headache is a common complaint presented in the practice of ophthalmologists and optometrists [48,49]. Migraine is a type of primary headache that has been associated with sodium chloride intake and withdrawal effects of excessive dietary salt, potentially related to fluid imbalances and idiopathic edema [50]. During migraine, sodium permeability increases in the blood–brain barrier and blood–cerebrospinal fluid barrier [51]. Importantly, reductions of between 31–46% in migraine incidence are associated with lower-sodium dietary patterns [52,53]. These findings imply that myopia associated with migraine headaches might also improve with lower levels of dietary sodium as fluid balance is restored within the vitreous.

Neurodegeneration and demyelination of the central nervous system in MS are associated with high sodium chloride intake, and higher sodium concentrations were detected in brain lesions and grey and white matter of the central nervous system in patients with MS [54,55]. Moreover, compared to people without MS, impaired visual acuity, such as blurred vision, is higher in patients with MS, even without a history of optic neuritis associated with MS [56]. Coincidently, a 2020 systematic review and meta-analysis of international studies found that the incidence of migraine headache is 31% in patients with MS, which is more than twice the migraine incidence in the global population [57]. Associations of migraine, MS, and myopia are potentially mediated by sodium chloride and require further investigations.

Importantly, potassium increases sodium excretion in the kidneys [58], but “even mild potassium depletion impairs the renal ability to handle a short-term sodium load” [59]. The WHO-recommended daily minimum intake for dietary potassium is 3500 mg, yet a 2022 systematic review and meta-analysis of 14 countries in the Americas found that mean potassium intake was 30% below WHO minimum recommendations [60]. The meta-analysis also found that mean sodium intake was approximately twice as high as WHO maximum recommendations, and the researchers suggested a need to promote a greater intake of fruits and vegetables, which are high in potassium and low in sodium. An imbalance of sodium and potassium, exacerbated by the increasing caloric intake of ultra-processed foods, which are low in potassium and high in sodium, could further contribute to increasing myopia rates.

## 4. Sodium Chloride, Osmolality, and Myopia

In cellular volume homeostasis, water flows across a cell membrane into a solution with a higher solute concentration, creating an osmotic gradient, and the number of solute particles in a solution determines the attraction of water, also known as the solution’s osmolality, by mass (solute particles per kg solvent) or osmolarity by volume (solute particles per L solvent) [61]. Hypertonic solutions have high osmolality, and hypotonic solutions have low osmolality. “All cells face constant challenges to their volume either through changes in intracellular solute content or extracellular osmolality” [61]. In ocular tissue, intravenous injections of hypertonic saline (containing higher concentrations of sodium chloride than in the blood) were observed to draw fluid out of the ocular vitreous and temporarily lower intraocular pressure, likely by increasing “the osmotic gradient between tissues and the blood, which pulls fluid from interstitial spaces to the intravascular space” [62]. 

Conversely, an osmotic gradient flowing in the opposite direction implies that fluid may be drawn into the vitreous through the increased ionic permeability of ocular membranes, such as in the retina pigment epithelium (RPE). For example, Bringmann et al. noted that “osmotic conditions at the basal side of the RPE regulate the tightness of the outer blood–retinal barrier; high osmolality increases and low osmolality decreases the permeability of the barrier” [63]. The researchers noted that “high salt and high extracellular osmolality have direct effects on RPE cells”, which include “alteration of the transmembrane sodium chloride gradient”. Furthermore, injections of normal saline (0.9% sodium chloride) into the vitreous of mouse eyes were found to dose-dependently damage the retina and RPE and upregulate inflammatory gene expressions of interleukin 6 (IL-6), interleukin 1-β, inducible nitric oxide synthase, tumor necrosis factor α (TNF-α), and vascular endothelial growth factor [64]. Cytokines are common in chronic inflammatory conditions, and IL-6 and TNF-α are among the cytokines upregulated in myopia progression [65], which is suggested to weaken and remodel scleral connective tissue [66]. Experiments by Zhang et al. on inflammation in RPE cells found that “high-salt intake may increase the risk of developing inflammatory diseases in the posterior segment of the eye. Further confirmatory studies are needed to investigate the effect of high-salt on cytokine production by primary human RPE cells” [67].

## 5. Vitreous Fluid Accumulation

The apical barrier of the RPE—the endothelial cells of the retinal blood vessels—has also been hypothesized to break down from hyperosmolar stress, leading to fluid and solute leakage from the retina and choroid (the vascular layer of the eye), with “massive water accumulation that can affect vision” [68]. For example, Cases et al. found that the liquid vitreous fraction in mice with high myopia was at least eight times higher than in control mice, while the sodium and chloride concentrations and osmolality in the myopic vitreous were slightly lower [69], possibly due to the diluting effect of excessive water volume. Additionally, experimental induction of myopia through vision deprivation (form-deprivation myopia) increased the volume of the liquid vitreous in neonatal chicks [70,71]. Marshall and Crewther posited that “water efflux across the RPE to the choroid has to be responsible for the concomitant change in the shape of the eye and changes in the fluid volume of the vitreous humor” [72]. Notably, “The fenestrated capillaries in the choroid are very permeable to low molecular weight substances; sodium permeability in the choroid is probably 50 times that in skeletal muscle” [73].

Earlier experiments in rabbits demonstrated that hypertonicity inside the vitreous barrier, subsequent to serum injections of urea, osmotically induced “an increase in the size of the vitreous body from an excess uptake of water to compensate for the existing hypertonicity” [74]. The researchers concluded that the vitreous volume “is not stable but is dependent, i.e., on the condition of the blood”. Furthermore, Kinsey described the ocular movement of sodium and chloride ions from the blood across the ciliary epithelium (iris blood vessels) into the posterior chamber and the diffusion of the ions between the posterior chamber and the vitreous [75]. In addition to affecting the vitreous fluid, a case of osmotic swelling in the aqueous humor-filled anterior chamber of a patient with hypernatremia was induced by sodium shifted into the lens, causing lens fiber damage and the rapid onset of myopic error [76].

Abnormal concentrations of sodium chloride in the vitreous of postmortem human cases were associated with antemortem hyponatremia and hypernatremia in the deceased patients, often in conditions with dysnatremia, such as community-acquired pneumonia [77]. Choroid thickening in patients is associated with increased blood plasma volume from water retention in hyponatremia [78], also known as hypervolemic hyponatremia [79]. Conversely, choroid thinning occurs in myopia as the ocular axis elongates [80], which stretches and thins the choroid [81], sclera [82], and retina [83]. Evidence suggests that extensibility of ocular tissue in myopia may play a role in helping to downregulate intraocular pressure through volume expansion. For example, intraocular pressure is lower in patients with medium myopia, compared to high myopia [84]. Importantly, thinning of ocular tissue from biomechanical changes in axial elongation should be differentiated from thinning that occurs due to neurodegeneration in conditions such as retinal endothelial cell apoptosis [85]. Coincidently, high dietary salt intake in a rat model of retinal ischemia/reperfusion exacerbated neurodegeneration of the retina, causing cell apoptosis and retinal thinning [86].

Age-related deterioration of the vitreous gel (liquefaction) is associated with the loss of viscoelasticity of the eye [87]. As ocular tissue loses elasticity, the inability to stretch and compensate for increasing pressure from fluid retention may increase the risk of ocular hypertension, which damages the retina and optic nerve in diseases like glaucoma [88]. Loss of viscoelastic biomechanical properties was found in an examination of human donor eyes with glaucoma, which were stiffer and less responsive to changes in intraocular pressure [89]. The interaction of impaired tissue extensibility with increased fluid volume and hypertension induced by high concentrations of sodium chloride could help explain the association of salt intake with open-angle glaucoma [90].

## 6. Future Myopia Research

In total, a synthesis of the evidence reviewed in this paper implies that fluid imbalance in myopia due to disturbances in osmolality might be reversed with a low-salt diet. For example, retinal vasculopathy involving the renin–angiotensin–aldosterone system (RAAS) was lowered through dietary salt restriction in laboratory animals [91]. Noting the high water content of the eye and the unique ocular system of fluid regulation, Australian authors conducted a 2015 systematic review and found that “systemic hydration status broadly affects a variety of ocular pathophysiologic processes and disease states” [92]. The authors linked hydration status specifically to the effect of sodium and the RAAS, and the authors recommended future research to investigate “acute and chronic changes in hydration in individuals with and without ocular disease”. Relatedly, axial length was significantly decreased, and intraocular lens power increased in 22 participants while fasting, compared to non-fasting periods, and a later study confirmed that alterations in axial length during fasting are attributed to changes in “the posterior segment of the eye”, which includes the vitreous humor [93]. Future studies should investigate the effects of fasting on axial myopia associated with reduced sodium chloride intake.

**Figure 4:**
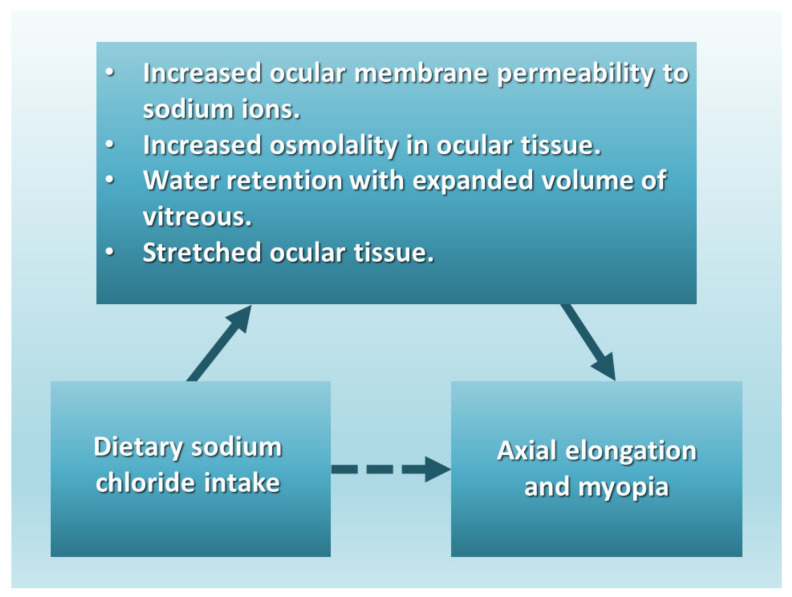
The proposed association of axial elongation and myopia with dietary sodium chloride intake is mediated by increased ocular membrane permeability to sodium ions, increased osmolality in ocular tissue, water retention with expanded vitreous volume, and stretched ocular tissue.

Myopia research studies using animal models have also demonstrated an emmetropization mechanism, “the developmental process that matches the eye’s optical power to its axial length so that the unaccommodated eye is focused at distance” [94]. However, animal models have not identified the underlying cause, prevention, or progression of myopic refractive errors [95]. Future myopia animal models should test different intake levels of dietary sodium chloride on axial length and emmetropization. Human intervention studies should test very low daily sodium intake (<200 mg) on reducing the cause of refractive errors, similar to the studies conducted by Walter Kempner [9]. The following directed acyclic graph (Figure 4) proposes that the association of sodium chloride intake with axial elongation in myopia is mediated by increased ocular osmolality, water retention with expanded volume of the vitreous, and stretched ocular tissue. 

## 7. Conclusions

Native people such as the Yanomamo tribe of Brazil have low prevalence of myopia and do not use salt in their diet. The increasing prevalence of myopia is predicted to affect approximately half of the global population by 2050. Theories of myopia etiology have neglected investigations of dietary sodium chloride. The present nutritional epidemiology perspective proposes that axial myopia occurs from increased fluid in the vitreous, induced by dietary sodium chloride. Salt increases the ionic permeability of retinal membranes and raises the osmotic gradient flow of fluid into the vitreous, which stretches ocular tissue during axial elongation. Experimental research should investigate the effect of dietary sodium chloride on axial elongation in myopia, and human dietary interventions should test a very low-salt diet in the correction and prevention of myopia.

## Data Availability

Not applicable.
